# *Helicobacter pylori* in oral and gastric pathologies: a narrative review of potential bidirectional pathogenic interactions

**DOI:** 10.1080/07853890.2025.2533434

**Published:** 2026-06-26

**Authors:** Erandis Dheni Torres Sánchez, Melissa Martínez Nieto, Gustavo Eder González Alvarez, Ruth Rodríguez Montaño, Mario Alberto Alarcón-Sánchez, Artak Heboyan, Adrián Fernando Gutiérrez Maldonado, Juan José Varela Hernández, Sarah Monserrat Lomelí Martínez

**Affiliations:** a Department of Medical and Life Sciences, La Ciénega University Center, University of Guadalajara (CUCIENEGA-UdeG), Ocotlán, Jalisco, Mexico; b Department of Integral Dental Clinics, University Center of Health Sciences, University of Guadalajara (CUCS-UdeG), Guadalajara, Jalisco, México; c Department of Health and Illness as an Individual and Collective Process, University Center of Tlajomulco, University of Guadalajara (CUTLAJO-UdeG), Tlajomulco de Zúñiga, Jalisco, México; d Institute of Research in Dentistry, Department of Integral Dental Clinics, University Center of Health Sciences, University of Guadalajara (CUCS-UdeG), Guadalajara, Jalisco, México; e Molecular Biology Department, University Center of Health Sciences, Universidad de Guadalajara (CUCS-UdeG), Guadalajara, Jalisco, México PhD Student in Molecular Biology and Medicine; f Department of Prosthodontics, Faculty of Stomatology, Yerevan State Medical University after Mkhitar Heratsi, Yerevan, Armenia; g Department of Medical and Life Sciences, Centro Universitario de la Ciénega, Universidad de Guadalajara, Ocotlán, Mexico

**Keywords:** Periodontitis, gastritis, *Helicobacter pylori*, periodontal disease, dental plaque

## Abstract

The association between periodontal diseases and gastrointestinal conditions, particularly those associated with *Helicobacter pylori* and systemic inflammation, has garnered increased scientific attention because of its clinical and public health implications. These diseases, which affect both the oral cavity and the digestive system, have shared pathophysiological mechanisms that link inflammatory processes and bacterial transmission pathways. The possible presence of *H. pylori* in the oral cavity has sparked interest regarding its potential colonization of periodontal tissues and acting as an extragastric reservoir. This narrative review describes *H. pylori*’s possible survival mechanisms in this oral microenvironment and its clinical significance in the interaction between oral and gastric conditions. We propose that periodontitis might promote gastric *H. pylori* infection by stimulating systemic inflammation, and oral colonization might serve as a reservoir for gastric reinfection. Future studies may involve advanced technologies such as metagenomics and proteomics. The eradication of *H. pylori* in the oral cavity may provide a strategy to prevent gastric reinfection. The findings described herein highlight the importance of this bacterium in two different pathologies sharing a close anatomical relationship.

## Introduction

*Helicobacter pylori* (*H. pylori*) infection is among the most important current challenges in medicine, given its major role in the etiologies of various gastric pathologies including peptic ulcers, chronic gastritis, and even gastric cancer [1–4]. The prevalence of this microorganism, coupled with its versatility to adapt to and colonize various niches of the human body, has made it a focus of both basic and clinical research. Historically, *H. pylori* was considered exclusively in the context of the gastrointestinal system; however, in recent years, the identification of its presence in the oral cavity suggests a potential extragastric reservoir that might promote digestive tract reinfection and hinder eradication protocols [3,5–7].

Periodontitis, a chronic inflammatory pathology of dental support tissues, involves a multifactorial process comprising interactions among pathogenic microorganisms, dysregulated immune responses, and environmental factors [8,9]. The systemic inflammatory process triggered by this condition enhances the dissemination of certain inflammatory mediators that compromise the integrity of the gastric mucosa, thus establishing a link between oral and gastric health. The coexistence of these two conditions, and their shared mechanisms of action, suggests potential bidirectional interactions, wherein oral colonization by *H. pylori* not only perpetuates a gastric infection but also triggers a more severe and destructive periodontal inflammatory process [3,5–7].

In this context, this narrative review is aimed at integrating and analyzing recent findings regarding the interaction between *H. pylori* infection and periodontal conditions. Fundamental aspects such as the etiology and pathophysiology of *H. pylori*-induced gastritis, transmission routes, virulence factors, and the involvement of the oral microenvironment in the persistence of the microorganism are described. Similarly, this review describes how microbial dysbiosis and the immune response create an environment conducive to *H. pylori* colonization and proliferation, thereby promoting the recurrence of gastric infections. Better understanding these pathological processes might influence the diagnosis and treatment of associated conditions, and lead to the development of comprehensive therapeutic strategies. Advanced techniques such as metagenomics and proteomics may offer new perspectives regarding the links between the oral and gastric microbiota, and facilitate the identification of risk biomarkers and the design of more effective interventions. Likewise, the eradication of *H. pylori* in the oral cavity might provide a complementary strategy for preventing gastric reinfection and decreasing systemic inflammatory load.

This review provides an updated global portrait of the association between *H. pylori* and periodontitis, highlighting the need for integrated approaches in the treatment of these conditions. Consolidation of advanced knowledge in this area is essential to optimize clinical outcomes and ultimately enhance patient quality of life.

## Search methodology

This narrative review was developed through a structured methodological strategy to ensure an adequate, rigorous, and well-founded selection of literature. An exhaustive search was conducted in the Scopus, PubMed, and Web of Science databases, including publications from the last 20 years. Publications in English or Spanish were included, comprising studies with observational or experimental designs, or reviews that analyze the presence, colonization, virulence mechanisms, or clinical interactions of *H. pylori* in the oral or gastric environment, as well as studies linking *H. pylori* colonization to relevant periodontal, gastric, or systemic diseases. Boolean combinations with specific terms were considered, such as: ‘*Helicobacter pylori*’ AND ‘oral cavity’, ‘gastric mucosa’, ‘periodontitis’, ‘bidirectional transmission’, ‘virulence factors’, ‘oral microbiota’.

## Periodontitis: etiology and pathophysiology

Periodontitis is a long-term inflammatory condition involving gradual destruction of the tissues supporting the teeth, such as the periodontal ligament and the bone surrounding the teeth. The disease is caused by a complex interaction of factors, including pathogenic bacteria, the body’s immune and inflammatory responses, and various environmental and genetic risk factors [8–10]. The dysregulated immune response and uncontrolled inflammation, influenced by both genetic and environmental factors, ultimately lead to the tissue destruction observed in the disease [11,12].

Genetic susceptibility plays a major role in the development of periodontitis. As much as 50% of an individual’s risk has been estimated to be associated with heredity [13]. Genetic factors can affect the immune response, inflammation, and loss of bone supporting the teeth [14].

Environmental risk factors, such as smoking and diabetes, increase the likelihood of developing periodontitis. Smoking impairs immune function, promotes inflammation, and significantly increases the risk of severe gum disease and tooth loss [9,11]. In addition, psychological stress might contribute to periodontitis by negatively affecting immune function and leading to poor oral hygiene habits [11].

The inflammatory process is central to periodontitis, by facilitating the spread of microorganisms from the oral cavity to other parts of the body. A key molecular mechanism underlying the tissue damage in periodontitis is the imbalance between pro-inflammatory and anti-inflammatory cytokines, which regulate immune responses and cell signaling [15]. Although the presence of bacteria is necessary for the disease to develop, the body’s immune dysfunction and chronic inflammation ultimately result in the tissue destruction characteristic of periodontitis.

## *Helicobacter pylori*-induced gastritis: etiology and pathophysiology

On the other hand*, H. pylori* is a Gram-negative aerobic bacterium 2–4 μm in length. Its 3 μm long flagella trigger alterations in the epithelial cells of the gastric mucosa and consequently lead to tissue destruction. In humans, the prevalent *Helicobacter* species are *H. pylori* and *H. heilmannii*, both of which trigger gastritis, dyspepsia, peptic ulcers, and gastric adenocarcinomas [1,2,16]. The gastric epithelium provides a favorable environment for the growth of this bacterium; nevertheless, the infection process depends on the individual’s immune response, in interaction with environmental variables [2].

This bacterium causes inflammatory alterations in the gastric mucosa and duodenum that depend on the type of *H. pylori* and its relationship with cytotoxic gene A (cagA). *H. pylori* infection can lead to hypochlorhydria, hypergastrinemia, decreased ghrelin and leptin levels, alterations in the gastrointestinal microbiota, and immune activation in affected individuals [4]. This bacterium catabolizes the conversion of cholesterol from gastric cells to cholesterol alpha glycoside, thus affecting the release of pro-inflammatory cytokines and activating the enzyme nitric oxide synthase 2, which is responsible for releasing cellular nitric oxide, a powerful oxidizing agent [17]. For *H. pylori* to thrive in the gastric mucosa, adequate functioning of the urease enzyme is required to create an environment conducive to its growth. Additionally, the bacterium requires the release of glycoproteins to enable the mobility of its flagella, as well as outer membrane proteins that facilitate adherence to the epitheliumTogether, these characteristics of *H. pylori* promote its growth [17,18]. The flagellar motility of this pathogen is enhanced by urea and bicarbonate in the mucosa, thus enabling penetration of layers with a neutral pH. *H. pylori* adheres to the ileum, colon, and biliary portions [1,16].

When *H. pylori* colonizes a cell, it increases the expression of cagA, an oncogene associated with the development of gastric adenocarcinomas. Subsequently, cagA exacerbates the release of pro-inflammatory cytokines and the overexpression of MMP types 2, 7, and 9. These MMPs in turn change the extracellular matrix, thus altering cell signaling, promoting the binding of bacteria to epithelial cells, and perpetuating the damage caused by this bacterium [18].

Individuals positive for *H. pylori* have a 10%–20% risk of developing ulcerative diseases and a 1%–2% risk of developing gastric cancer. Colonization by this pathogen begins with acute gastritis, which can become chronic and lead to hypochlorhydria, increased bacterial colonization and persistent inflammation in the mucosa. If this colonization is uncontrolled, peptic ulcers with mucosal damage of 0.5 cm or more in diameter may form [16].

## Epidemiology and transmission: global prevalence and transmission routes

The prevalence of *H. pylori* is particularly pronounced among inhabitants of developing countries and certain ethnic groups. Countries such as Ghana, Morocco, Turkey, China, Nigeria, Portugal, Estonia, Kazakhstan, Pakistan, Russia, Jordan, Iran, Canada, and various Latin American countries notably have population colonization rates exceeding 51%, and adults and older people are the main groups affected. The presence of *H. pylori* has also been associated with low socioeconomic levels, thus posing health risks in this demographic. In the year 2021, 4.4 billion individuals with *H. pylori* were reported worldwide; however, specific findings by sex subgroup were not reported [1,16,19].

Li and collaborators have reported a decrease in the prevalence of infection with this pathogen from 58.2% between 1980 and 1990 to 43.1% between 2011 and 2022 in 71 countries, according to data from the World Health Organization. This decrease might be associated with an expansion in universal health coverage [20]. A meta-analysis conducted from 1980 to 2022 by Chen’s research team has reported similar findings in adults, whereas children and adolescents showed no decrease in prevalence. The largest decrease in prevalence was observed in countries in the Western Pacific, South Asia, and Africa. Importantly, the decline in prevalence was associated with a decrease in gastric cancer incidence in the population, particularly in countries such as Brazil, China, Greece, Japan, and South Korea. Improved food storage conditions might potentially have mitigated the proliferation of *H. pylori*, which spreads more rapidly in unhealthful conditions [1,21].

To date, forms of *H. pylori* infection have not been clearly described; however, the transmission routes have been found to include the fecal-oral route, oral-oral route, food or water ingestion, the gastric-oral route, breastfeeding, iatrogenic routes, zoonoses, and even sexual contact. The most relevant routes might be fecal-oral and oral-oral transmission. This pathogen has been found to demonstrate horizontal transmission, through interactions among people not linked by blood or through consumption of contaminated food or water. In contrast, vertical transmission involves intergenerational pathogen transfer among blood relatives. Among the *H. pylori* risk factors, living in areas of low socioeconomic status is particularly notable. Another contributing factor is the consumption of food prepared under poor hygiene conditions. Additional risks arise from diagnostic or invasive treatments, genetic predisposition, and contact with *H. pylori*-infected individuals, particularly among clinical personnel. The risk also increases in early childhood and as age progresses [1,16,19,22,23]. The risk of contagion by this pathogen is particularly high during the first month of life, because of contact with positive pregnant mothers [19].

## Pathogenic mechanisms: roles of vacA and cagA, urease production, and gastric mucosal damage

Several mechanisms of pathogenicity by *H. pylori* have been described. First, this bacterium’s high urease activity allows it to metabolize urea to ammonium and carbon dioxide. In fact, 6%–10% of *H. pylori* proteins are ureases. The increase in ammonia ions and bicarbonate shifts the acid-base balance toward a more alkaline state facilitating colonization by this bacterium at a pH below 4, the bacterium loses mobility. In parallel, ammonia ions exert a cytotoxic response in epithelial cells, whereas bicarbonate decreases the bactericidal activity of nitric oxide and promotes the release of lipopolysaccharides. Subsequently, the lipopolysaccharides alter the gene expression of the endothelial mucosa through post-transcriptional and post-transductive changes, thereby triggering chronic inflammation of the tissue and regulating the interaction of antigens with the glycoproteins of the gastric epithelium through adhesins, and exacerbating gastric mucosal lysis. After *H. pylori* enters epithelial cells, it induces the activation of protease enzymes that lyse the lipase enzymes of the epithelium, thus degrading important phospholipids that form the protective layer of the gastric mucosa [1,16,24]. Lipopolysaccharide release leads to an increase in pro-inflammatory cytokines such as interleukin-1 beta (IL-1β) and IL-8, and the release of free radicals [1].

In addition, *H. pylori* mediates the release of exotoxins such as cagA and vacuolating cytotoxin A (VacA), which are involved in gastric mucosa inflammation and ulceration [25]. When *H. pylori* invades the host, it stimulates increased cagA gene expression. The CagA protein acts on the phosphorylation of tyrosine through Src-C kinases in amino acids, such as Glu, Pro, Ile, Tyr, and Ala, within EPIYA motifs; it also activates other proteins such as SHP-2, Crk, Grb2, ZO-1, and JAM. The relationship between cagA and this series of enzymes and protein factors promotes cell motility, by damaging the tight junctions of epithelial cells, decreasing adhesion between the cells, and finally destabilizing the epithelial membrane. The stability of CagA and its regulation of phosphorylation depend on CagF, which promotes the stability of CagA, and CagL, which promotes the binding of CagA to integrins of epithelial cells. Additionally, CagA stimulates the transcription factor NF-κB and T cells associated with IL-8 release. Finally, CagA is associated with DNA damage and endothelial cell apoptosis through increased oxidative stress [1,16,27].

VacA proteins interact with adhesins of the epithelium and facilitate their vacuolization, thus leading to the formation of pores in the epithelium and in the mitochondrial membrane and affecting cellular apoptosis [1,27]. VacA stimulates the formation of membrane channels, alters endosome trafficking, and influences lysosomal activity, thereby decreasing autophagy; it also induces changes in the cellular cytoskeleton and in the immune response. The formation of pores in the epithelium induces the release of urea, which requires *H. pylori* to modify the acid-base balance of the gastric mucosa, in addition to the rupture of the tight epithelial junctions. Similarly, the increase in VacA protein is associated with inhibition of T cell proliferation. Thus, VacA is important in *H. pylori* proliferation in the gastric mucosa [16,27,28].

The relationship between CagA and VacA in the host has also been studied. VacA promotes the expression and phosphorylation of the CagA gene. The pathogenicity of CagA may depend on the activity of VacA; CagA is rapidly degraded in the absence of VacA, which increases the half-life of CagA. However, VacA and CagA have been reported to have antagonistic effects [27,28]. However, precise data regarding this relationship remain lacking.

## Presence of *H. pylori* in the oral cavity

Given that the oral cavity is the entrance and the first element of the gastrointestinal system, its role as a possible reservoir for *H. pylori* has been debated. A possible oral-oral transmission route of *H. pylori* prompts questions regarding whether close contact, might facilitate *H. pylori* spread [29].

*H. pylori* was first detected in dental plaque in 1989 [30]; consequently, plaque was considered a potential reservoir for this bacterium, particularly in periodontal patients. The environment in the periodontal pocket is conducive to growth of this microaerophilic bacterium. Thus, it is considered a link between periodontal pathology and stomach infection by *H. pylori* [6]. *H. pylori* is found in saliva, at the back of the tongue, and in dental plaque; however, the oral cavity does not appear to provide an optimal environment for *H. pylori*, because of thermal instability, high levels of O_2_, and diverse bacterial composition [31].

The environment of the oral cavity differs considerably from that of the stomach [32]. For example, Streptococcus mitis and Streptococcus mutans may inhibit *H. pylori* growth *in vitro* [33]. However, *H. pylori*, particularly in supragingival plaque, has been found to be a risk factor for periodontitis. In one study, the total percentage of periodontal pathogens in oral subgingival plaque samples with *H. pylori* positivity exceeded those that were *H. pylori* negative [34].

These findings clearly indicate a relationship between periodontitis and gastritis induced by *H. pylori*. However, studies evaluating the presence of *H. pylori* in the mouth have yielded contradictory findings. Agarwal and Jithendra et al. have found *H. pylori* in subgingival plaque samples from 18 (60%) patients with confirmed gastric *H. pylori* infection [35]. Dane and Gurbuz have examined dental plaque from 35 patients with gastric *H. pylori* infection by using the CLO gel test and found oral *H. pylori* in 29 (82.8%) patients [36]. In contrast, the traditional cultivation method has indicated several discrepancies in the identification of *H. pylori* [6,37].

## Potential mechanisms of pathogenic interaction

*H. pylori* adapts to the oral environment by invading periodontal ligament fibroblasts, similarly to how it invades gastric epithelial cells in the stomach. This invasion enables *H. pylori* to complete its life cycle within the oral tissues, thus disrupting cellular processes and causing localized tissue damage. By invading these fibroblasts, *H. pylori* evades immune defenses and survives in an environment less favorable than the stomach. This ability to invade and persist in both gastric and oral environments highlights the bacterium’s remarkable adaptability and potential to contribute to disease progression at both sites [5].

**Figure 1.**
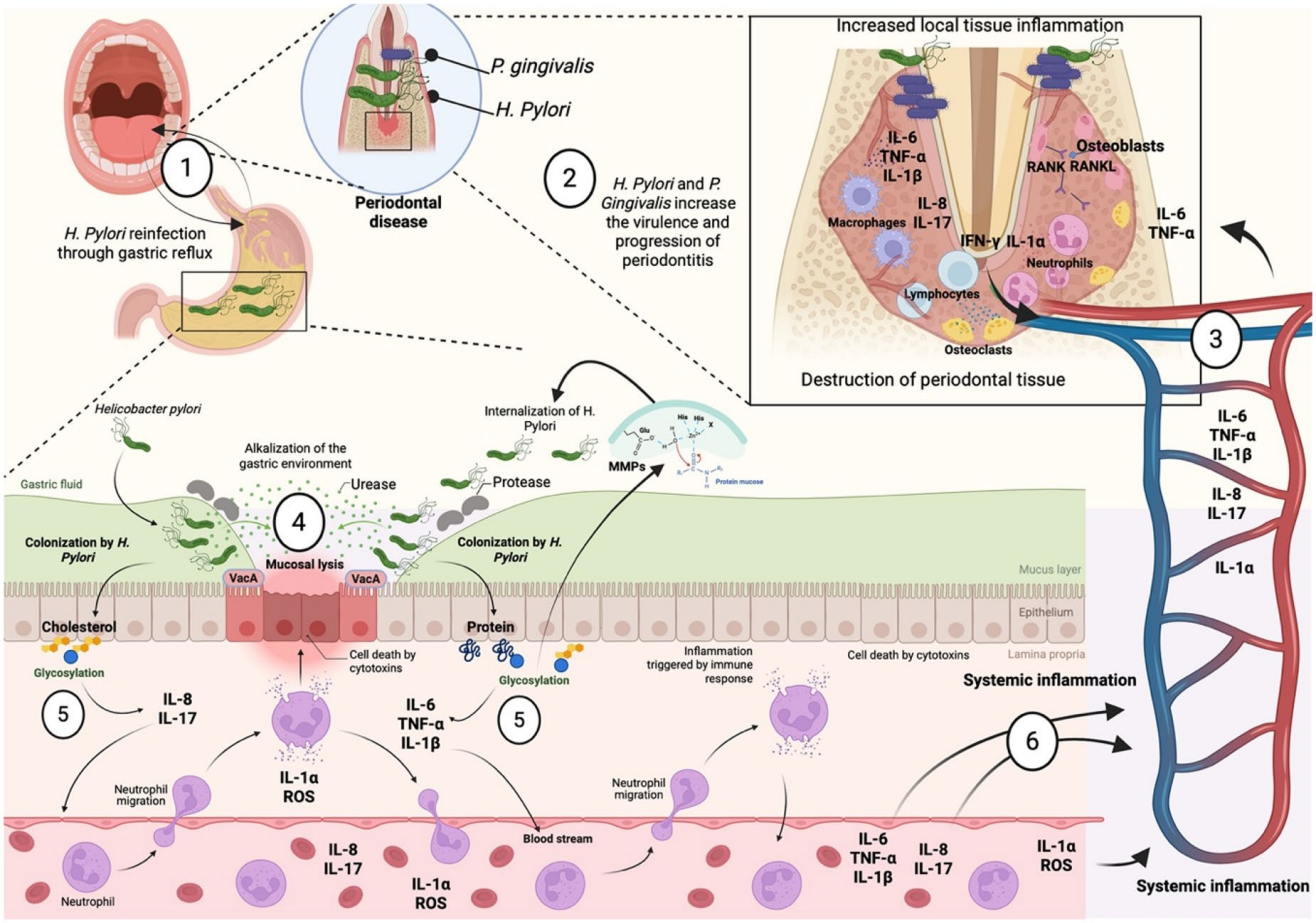
Bidirectional mechanism of *H. pylori* between the oral cavity and the gastric mucosa. 1) The oral cavity is a reservoir of *H. pylori*, enhancing the colonization of bacteria that cause periodontal disease. 2) Bacterial colonization triggers an inflammatory response that promotes tissue destruction. 3) The increased oral inflammatory response irrigates the systemic circulation. 4) *H. pylori* in the gastric mucosa modifies its own architecture through different mechanisms, damaging the mucosa and facilitating colonization by *H. pylori*. 5) *H. pylori* by glycosylation contributes to the increase in the inflammatory and oxidative response. 6) Inflammatory markers irrigate the systemic circulation, feeding back the cycle. Created in BioRender. Torres, D. (2025) https://BioRender.com/evw3bd2.

The presence of *H. pylori* in the mouth is typically temporary in asymptomatic individuals, and often occurs after episodes of vomiting or acid reflux. The bacteria can travel from the stomach to the oral cavity, and may colonize dental plaque and nasopharyngeal tissues (Figure 1). Such gastro-oral transmission is frequently observed, particularly in patients with gastroesophageal reflux disease, in which gastric acid carries *H. pylori* into the mouth, thus allowing it to persist in areas such as dental plaque and tonsils [38,39].

In the oral cavity, *H. pylori* triggers an immune response that can contribute to periodontal disease progression. The bacterium increases the expression of interleukin (IL)-17, a pro-inflammatory factor with a key role in periodontal tissue destruction. In people with periodontitis, diminished methylation of the IL-17C promoter region leads to elevated levels of IL-17, which mediates inflammation and tissue damage [40].

IL-17 further promotes bone resorption in the periodontal tissues by regulating RANKL and osteoprotegerin, key molecules involved in bone remodeling. This pathway contributes to the progressive destruction of periodontal bone and ultimately leads to the bone loss observed in periodontitis. Furthermore, *H. pylori* colonization in the stomach can amplify IL-17 expression and potentially aggravate inflammatory damage in periodontal tissues. Thus, by promoting IL-17 expression, *H. pylori* can worsen periodontal tissue destruction by contributing to both bone loss and the degradation of connective tissues in the gums [40].

## Dynamics of systemic inflammation and the immune response

*H. pylori* can colonize the epithelium of the gastric glands [41]. Through chemotaxis, this bacterium evades toxins such as reactive oxygen species and takes up nutrients including urea, arginine, cobalt, nickel, iron, and even lipids from host cells [42,43]. TlpA receptors recognize arginine, which helps the bacterium grow, whereas TlpB receptors sense urea and gastric mucus [44,45].

Some virulence factors of *H. pylori* are part of the broad Hop family of outer membrane proteins (OMPs). The most recognized adhesins of *H. pylori* are BabA, SabA, AlpA/B, HopZ, and OipA, all of which belong to the Hop family [43].

Strains positive for blood group antigen-binding adhesion (BabA) have been found to colonize with greater density and to induce a more intense secretion of IL-8 in the mucosa than strains with insufficient BabA [46]. Moreover, outer inflammatory protein A (OipA) increases the secretion of IL-8 from gastric epithelial cell lines [47]. Similarly, lipoproteins associated with AlpA and AlpB adhesion induce IL-6 and IL-8 production in gastric cell lines [48]. Moreover, the adherence of *H. pylori* to the extracellular matrix protein laminin is mediated by SabA, a sialic acid-binding adhesin [49].

After *H. pylori* enters the mucous layer after a brief period within the gastric lumen, it spreads approximately 25–30 μm from the mucosal epithelial cells, where the pH varies from 4.5 to 6.5; it has also been found directly in the epithelium or deep within the glands [50].

*H. pylori* in the oral cavity promotes periodontal disease by modifying the microecology. The pre-incubative interaction between *Porphyromonas gingivalis* (*P. gingivalis*) and *H. pylori* influences the virulence of *P. gingivalis* through the creation of biofilms, the internalization of bacteria in oral keratinocytes, and hemagglutination. Consequently, direct interaction between *P. gingivalis* and *H. pylori* in the subgingival plaque increases the severity or progression of periodontitis [51]. Additionally, the expression of the Wnt5a protein, which is associated with periodontitis, and the cytokines IL-8, IL-6, and INF-γ, considerably increases after human leukemia mononuclear cell lines are stimulated with cagA+ *H. pylori*, thus indicating that *H. pylori* can worsen the progression of inflammation [34].

## Clinical coincidence vs. pathogenic bond

In recent years, the association between *H. pylori*, a microorganism with roles in gastric conditions, and its presence in the oral cavity has become a matter of clinical interest. The oral cavity has been identified as a potential extragastric reservoir for *H. pylori*, which might contribute to both gastric reinfection and periodontal conditions; this bidirectional association has consequently been explored [6].

Although primary infection is frequently triggered by fecal-oral or gastro-oral routes, the oral cavity plays a fundamental role as an extragastric reservoir that contributes to perpetuating recurrent infections [6]. In a descriptive study by Moosavian et al. [52], among 106 patients with dyspepsia, *H. pylori* was present in dental biofilm samples in 17.9% of patients and in gastric tissue in 52.8% of patients, although the genetic patterns differed in some cases. The authors concluded that no direct relationship exists between oral and gastric colonization by *H. pylori*. This result suggests that the oral cavity is an ecosystem that serves as a secondary source of transmission to the stomach, particularly after incomplete treatment. Consequently, the persistence of *H. pylori* in dental biofilm might hinder eradication of the microorganism in the gastric system; this possibility is particularly relevant in patients experiencing gastritis recurrence after the termination of eradication treatment. In contrast, in Iran, in an investigation in 67 patients with periodontitis, 23 of whom had gastritis, *H. pylori* was identified in 5.97% of dental biofilm samples and in 17.39% of patients with gastritis. The findings indicated a statistically significant association between the presence of the bacteria in dental biofilm and gastritis (*p* = 0.012). However, despite the low biofilm prevalence, dental biofilm can act as a reservoir for gastric reinfection by *H. pylori* [53]. These findings are similar to those reported by Ding et al. [54] and Yang et al. [4]. The latter study used a case-control design comprising cases with periodontitis and controls without periodontal conditions, and included 212 non-smoking adults. The patients harboring *H. pylori* had a 2.82 times higher risk of developing periodontal disease after adjustment for factors such as age, physical activity, body mass index, diabetes mellitus, and alcohol consumption, thus suggesting a significant association between *H. pylori* and periodontal disease. In the same context, Ding et al. [54] investigated the relationship between *H. pylori* infection in saliva and frequent oral pathologies (caries and periodontal disease) in 1,050 Chinese adult participants, by using salivary antigen tests. The prevalence of the bacterium was 60.29%, and the highest rates were observed in patients with caries (66.91%) and periodontal disease (63.42%), in contrast to healthy patients (54.07%). These findings demonstrated that oral infection by *H. pylori* was significantly associated with these conditions (*p* < 0.05) and might act as an extragastric reservoir. That study reported a much higher prevalence of *H. pylori* in oral samples (60.29%) than observed by Moosavian et al. [52] and Eskandari et al. [53] (17.9% and 5.97%, respectively). However, this discrepancy might be attributable to population or methodological differences: the first three studies used PCR, whereas the last study used salivary antigen tests, thus potentially influencing the findings.

Periodontitis, characterized by inflammation in the periodontal tissues generated by a dysbiotic microbiota, can exacerbate systemic inflammation and consequently affect the gastric mucosa through release of inflammatory mediators. Byun et al. [55], in a study in South Korea, used a cross-sectional design to obtain data from the Korean Genome and Epidemiology Study recorded from 2004 to 2016; 173,209 participants were analyzed, of whom 9,983 had periodontitis and 125,336 did not. The patients with periodontitis had a significantly higher risk of developing chronic gastritis (adjusted OR = 2.22, CI = 2.10–2.34) and gastric ulcers (adjusted OR = 1.86, CI = 1.74–1.98), even after adjustment for confounding factors such as obesity, smoking, and alcohol consumption. These findings support a hypothesis in which the systemic inflammatory condition generated in the oral cavity by the presence of periodontitis negatively affects gastric health, probably by altering the mucosal defenses against infections caused by microorganisms such as *H. pylori*. However, despite the large sample, the study’s reliance on self-reported data and lack of direct determination of *H. pylori* presence might potentially have led to an underestimation of the relationship.

Some studies have focused on the search for specific genotypes of *H. pylori* present in the oral cavity and stomach, and explored their epidemiological and pathogenic relationships. In oral and gastric samples, the cagA and vacA virulence genes have frequently been associated with elevated pathogenicity in strains. In a study in a Mexican population, the cagA gene was present in 21.7% of *H. pylori* strains isolated from dental biofilm, thus suggesting that highly virulent strains can colonize both ecosystems and increase both gastric and buccal inflammation; additionally, a significant correlation was found between the Periodontal Screening and Recording index and oral *H. pylori* infection (*p* < 0.05) [56] however, a direct relationship between cagA and periodontitis severity was not observed. Consequently, other local factors, such as the presence of periodontopathogenic bacteria and inflammatory mediators, might play a greater role in periodontal pathogenesis. Despite the relevance of the data obtained from the study, the relatively small sample of 38 patients limits the generalizability of the results; therefore, research with larger samples from different areas is necessary to confirm these observations. In contrast, Valadan et al. [57] have identified *H. pylori* DNA in 5% of dental biofilm samples; the prevalence was 8% in the periodontitis group and 2% in the group without periodontitis. However, a statistically significant correlation between the presence of *H. pylori* and periodontitis (*p* > 0.05) was not reported.

A meta-analysis by Chen et al. [7] has highlighted *H. pylori* as a fundamental factor in the link between periodontitis and gastric conditions; this study emphasized that patients with dyspepsia have elevated periodontitis risk when *H. pylori* is identified in the oral cavity. Among risk factors, poor oral hygiene was found to promote the accumulation of dental biofilm and to facilitate *H. pylori* colonization. Although not all studies controlled for these variables, the findings emphasize potential effects of tobacco and alcohol consumption on the progression of both periodontitis and gastric infections. The limitations of the analysis included the diversity of methods for diagnosing *H. pylori* infection and the clinical criteria for diagnosing periodontitis; in addition, the predominance of studies conducted in developing countries might have biased the findings toward communities with high rates of *H. pylori* infection. In contrast, López et al. [58] have highlighted the relevance of systemic inflammatory processes in linking periodontitis and gastric infection; additionally, the exacerbated immune response in periodontitis might contribute to the appearance of gastritis caused by *H. pylori*. The identified risk factors included advanced age, which leads to changes in microbiota and immune response in older adults with periodontitis, and systemic comorbidities, such as diabetes and cardiovascular diseases, which aggravate the association between gastritis and periodontitis. Although more than 200,000 participants were included in the analysis, some individual investigations had small sample sizes, thereby influencing the robustness of the findings. Additionally, no conclusive results indicated whether the presence of *H. pylori* in the oral cavity is a direct cause of gastritis or is simply an associated marker. Recently, Maurotto et al. [6] have concluded that *H. pylori* can colonize the dental biofilm independently of the stomach and vice versa; however, in the context of periodontitis and gastritis, its presence might be more significant. Because this bacterium can form a reservoir in the dental biofilm, people with gastric infections might have elevated likelihood of *H. pylori* presence in the oral cavity. The study highlighted the roles of certain factors increasing susceptibility to periodontitis, such as low socioeconomic status associated with lower access to oral services, and inadequate eating habits that foster an altered microbiota amenable to colonization by *H. pylori*. Likewise, the variability in detection methods (PCR, urease tests, and cultures) complicated comparisons with other studies, and integrated investigations over long periods led to variations in prevalence. These aspects are considered limitations of the study. In these meta-analyses, the association between gastritis and *H. pylori* was clearly evident; however, some discrepancies were found in the identified risk factors, methodological limitations, analytical approaches, and study population characteristics.

These studies provide valuable insights advancing current knowledge. However, they differ in methodological designs, the characteristics of the populations studied, and the diagnostic criteria, while agreeing on the determinants of the discrepancies observed. Therefore, additional studies with unified methods must be designed to clarify this relationship.

## Critical evaluation of the literature

The currently available scientific evidence on the link between *H. pylori* and periodontal diseases provides valuable insights that advance current knowledge. However, several interesting methodological limitations are evident that must be considered when interpreting the results of this narrative review.

First, there is a discrepancy in the various designs of the integrated studies, ranging from cross-sectional studies to case-control studies and cohorts, compromising the level of methodological rigor. This inconsistency influences the comparability of the reported findings and restricts their generalization to different populations.

Secondly, heterogeneity in diagnostic methods is significant. Both oral and gastric samples for the identification of *H. pylori* were obtained using various techniques, including PCR, urease tests, bacterial cultures, and salivary antigen tests, each with differing levels of sensitivity and specificity. These inconsistencies make it difficult to draw solid conclusions about the prevalence and patterns of colonization.

Third, population diversity presents a limitation. Most studies were conducted in Latin America and Asia, exhibiting a high baseline prevalence of *H. pylori* and limited oral health care. This is relevant because the epidemiological conditions, diagnostic methods available, and associated risk factors vary considerably among different geographical areas; consequently, we encounter biases and limitations when trying to extrapolate the findings to scenarios such as Europe, Africa, or North America, where lifestyles, health contexts, and sociodemographic profiles differ substantially.

Additionally, few studies correctly control for confounding factors such as socioeconomic level, oral hygiene habits, the presence of systemic conditions, and dietary habits. These factors impact both periodontal condition and susceptibility to colonization by *H. pylori*. Likewise, small sample sizes and a lack of follow-up complicate the determination of long-term links or causal relationships.

Finally, most studies are analyzed based on observational data without considering the validation of pathophysiological mechanisms; on the other hand, few studies have comparatively analyzed the genetic or virulence profiles of *H. pylori* in gastric and oral samples.

## Bidirectional mechanism of oral and gastric colonization

This bidirectional relationship has not yet been fully elucidated. The impact of periodontitis on *H. pylori* colonization and its consequences at the gastric level is the focus of most frequently published evidence. By contrast, he effect of *H. pylori* on periodontitis is reported in the literature to a lesser extent. In summary, the oral cavity is an extragastric reservoir of *H. pylori*, as previously described, this pathogen is located in different anatomical sites. Thus, this bacterium is a common denominator between periodontitis and gastritis [7,28–31]. *H. pylori* is a risk factor for the progression of periodontal disease, as well as damage to the gastric mucosa and its respective consequences [4,34]. The question that remains to be answered is how this interaction takes place.

To answer this question, the following points are highlighted:

1. *H. pylori* can be transmitted in more than one way, and the oral cavity is the common setting for all of them [16,19]. The process of gastric reflux serves as a gastro-oral route, facilitating the movement of *H. pylori* from the stomach to the mouth and vice versa, this perpetuates the reinfections of both sites with their respective colonizations [6].
2. Once *H. pylori* is in contact with the oral cavity, this pathogen invades fibroblasts of the periodontium. This interaction promotes the release of cytokines, including IL-6, IL-8, IL-17, and IFN-γ. These cytokines increase inflammation in oral tissue and contribute to the destruction of periodontal tissue [5,40]. This increased virulence and progression of periodontitis can be explained by the weakening of the epithelial barrier. And therefore internalization of bacteria in oral keratinocytes and hemagglutination, as reported in the literature [52].
3. The increased inflammation described above not only affects the oral level but also spreads systemically. This systemic inflammatory factor contributes to infection and reinfection by this pathogen at the gastric level, thereby exacerbating gastric mucosal damage [3,5–7,10]. A proinflammatory environment facilitates the spread of *H. pylori* from the oral cavity to the stomach because it finds damaged mucosa, which makes colonization easier [15].
4. Once in the gastric mucosa, *H. pylori* prepares the acidic environment to an alkaline medium through its urease enzymes. This alkalinity promotes the colonization of the gastrointestinal mucosa by this bacterium [1,16,24]. It is important to remember that ureases are responsible for releasing ammonia and bicarbonate. Both are involved in an increase of cytotoxins and lipopolysaccharides, which favor the release of IL-1β, IL-8, and free radicals. In synergy, *H. pylori* urease and protease enzymes promote gastric mucosal lysis [1,16,24].
5. On the other hand, in the gastric mucosa, *H. pylori* glycosylates cholesterol and proteins, which promote a proinflammatory and oxidative environment. This overexpresses MMPs. MMPs modify mucosal architecture. They also facilitate *H. pylori* internalization [16–18]. Furthermore, VacA exacerbates inflammation by stimulating NF-κB, T cells, IL-1β and IL-8, leading to the formation of gastric mucosal ulcers [1,27].
6. Once located at this point in the stomach: *H. pylori* exacerbates both reflux and the increase in inflammatory markers at the systemic level. This feeds back into the inflammation of the epithelial cells of the oral cavity, exacerbating the periodontal damage [5,6,40]. This perpetuates the bidirectional cycle described in point 1 (Figure 1).

## Clinical implications and practice recommendations

Current findings suggest a bidirectional association between periodontitis and *H. pylori*-induced gastritis [7]. The oral cavity might serve as an extragastric reservoir for this microorganism, thereby promoting gastric reinfection after incomplete treatment [6]. The presence of this bacterium in the dental biofilm not only perpetuates the periodontal inflammatory process but also might negatively influence the effectiveness of *H. pylori* eradication treatments at the gastric level [7,53]. Consequently, early detection and effective management of periodontitis in patients with *H. pylori* gastritis are fundamental to stopping the reinfection cycle and decreasing the systemic inflammatory load.

Effective management requires a multidisciplinary approach integrating both periodontists and gastroenterologists. Periodontal evaluation protocols are recommended in the routine care of patients with clinical conditions associated with *H. pylori* gastritis. Methods such as PCR may be used for detecting *H. pylori* in the dental biofilm, thus enabling the identification of active oral reservoirs and guiding the selection of specific antimicrobial treatment schemes for both the oral cavity and the gastric tract. Additionally, adequate oral hygiene instructions and regular control of oral biofilm through dental prophylaxis might serve as effective complementary strategies to prevent gastric reinfection.

In this framework, routine cross-consultations are recommended as part of a multidisciplinary approach in which gastroenterologists refer patients with *H. pylori* gastritis to a periodontist for comprehensive evaluation. Likewise, these specialists might consider the possibility of gastric infection in patients with periodontitis who have severe clinical conditions and multiple risk factors. Close collaboration among specialists would offer multiple benefits, such as improved *H. pylori* eradication, decreased recurrence of gastritis, and significantly diminished systemic inflammatory processes. A correct and effective periodontal intervention would not only eliminate the oral reservoir of *H. pylori* but also decrease the systemic inflammatory mediators that exacerbate gastric conditions.

Continued joint collaboration among specialists would promote early detection and effective treatment of periodontitis, thus constituting fundamental pillars for the comprehensive management of patients with gastritis due to *H. pylori*. The implementation of these strategies has potential to transform current approaches by improving clinical outcomes and decreasing the prevalence of these conditions in the population.

## Future perspectives

The association between *H. pylori* and oral and gastric conditions raises certain questions that still need clarification. As the possibility of the oral cavity serving as an extragastric reservoir becomes consolidated through scientific evidence, new lines of research emerge aimed at understanding the specific mechanisms that promote its persistence in various oral niches and its contribution to gastric reinfection.

The role of *H. pylori* as an extragastric oral reservoir opens a range of research possibilities that demand innovative and multidisciplinary approaches. To achieve advances in understanding this interaction, it is essential to design longitudinal studies that integrate comparative analysis between oral and gastric strains, implementing advanced molecular techniques to identify the virulence profiles and genetic similarities of both ecosystems.

The oral microbiome plays a primary role in the homeostasis of the oral cavity and directly affects *H. pylori*’s ability to colonize this ecosystem. Consequently, incorporating metagenomic analyses might be beneficial to determine the interactions between *H. pylori* and other specific oral microorganisms, such as *P. gingivalis*, *Tannerella forsythia*, or *Fusobacterium nucleatum*, as well as to identify how these interactions limit or promote the persistence of *H. pylori* in certain oral niches.

A more comprehensive understanding of the bidirectional interaction between *H. pylori* and its direct effects on oral and gastric health might lead to modifications in clinical practice guidelines to integrate the evaluation of oral health in patients with recurrent gastric conditions or infections. Likewise, strategies aimed at eradicating *H. pylori* in populations with a high prevalence of associated pathologies should be designed.

These research perspectives should not only enhance understanding of *H. pylori* but also contribute to the design and planning of comprehensive and effective therapeutic approaches, thereby enhancing patients’ quality of life, and improving resources in the clinical and public health fields.

## Conclusion

The analyzed findings support the hypothesis that *H. pylori* is not confined to the gastric niche but also can colonize the oral cavity, thus forming an extragastric reservoir that facilitates reinfection and perpetuates systemic inflammatory conditions. This bidirectional phenomenon, in which gastritis and periodontitis interact through combined mechanisms of inflammation and microbial dysbiosis, underscores the importance of comprehensively addressing both conditions in clinical practice. Despite the limitations posed by various factors such as diagnostic methods, sample sizes, and study populations, the findings emphasize that *H. pylori* eradication should include not only gastric treatment but also specific strategies for the oral cavity. The emerging planning, development, and application of innovative and advanced techniques show promise in enhancing understanding of pathogenic mechanisms and establishing diagnostic biomarkers to provide clinical benefit. Therefore, longitudinal and multidisciplinary studies must be designed to elucidate the connection between oral colonization and gastric infection, and subsequently optimize therapeutic strategies and improve quality of life for patients.

## Data Availability

Data sharing is not applicable to this article as no data were created or analysed in this study
